# Extracellular Vesicles Carrying miR-887-3p Promote Breast Cancer Cell Drug Resistance by Targeting BTBD7 and Activating the Notch1/Hes1 Signaling Pathway

**DOI:** 10.1155/2022/5762686

**Published:** 2022-05-23

**Authors:** Bing Wang, Yueping Wang, Xuedong Wang, Juan Gu, Wenyong Wu, Huaiguo Wu, Qingping Wang, Daoping Zhou

**Affiliations:** ^1^Department of Medical Laboratory Science, Fuyang Cancer Hospital, Fuyang, Anhui 236000, China; ^2^Department of Medical Laboratory Science, Anhui No. 2 Provincial People's Hospital, Hefei, Anhui 230041, China; ^3^Department of Molecular and Cell Biology, University of Connecticut, Storrs, CT 06269, USA; ^4^Department of Pathology, The Fifth People's Hospital of Wuxi, Nanjing Medical University, Wuxi, Jiangsu 214000, China; ^5^Department of Oncology, Anhui No. 2 Provincial People's Hospital, Hefei, Anhui 230041, China

## Abstract

**Objective:**

Chemoresistance remains the primary reason threatening the prognosis of breast cancer (BC) patients. Extracellular vesicles (EVs) contribute to chemoresistance by carrying microRNAs (miRNAs). This study investigated the mechanism of miR-887-3p mediated by EVs in BC cell drug resistance.

**Methods:**

MDA-MB-231-derived EVs were extracted and identified. BC cells were treated with different concentrations of doxorubicin, cisplatin, and fulvestrant, and the cell survival was evaluated. PKH26-labeled EVs were cocultured with BC cells, and the uptake of EVs was observed. miR-887-3p expression in BC cells and EVs was detected. After silencing miR-887-3p in MDA-MB-231 cells, BC cells were treated with EV-inhi to observe drug resistance. The target gene of miR-887-3p was predicted and verified. The levels of downstream Notch1/Hes1 pathway were detected. Xenograft experiment was conducted to evaluate the effect of EVs on the growth and drug resistance *in vivo*.

**Results:**

MDA-MB-231-derived EVs enhanced the drug resistance of BC cells. EVs carried miR-887-3p into BC cells. miR-887-3p expression was elevated in BC cells and EVs. miR-887-3p inhibition reduced the drug resistance of BC cells. miR-887-3p targeted BTBD7. Overexpression of BTBD7 partially reversed the drug resistance of BC cells caused by EV treatment. EV treatment increased the level of Notch1/Hes1, and overexpression of BTBD7 decreased the level of Notch1/Hes1. *In vivo* experiments further validated the results of *in vitro* experiments.

**Conclusion:**

EVs carrying miR-887-3p could target BTBD7 and activate the Notch1/Hes1 signaling pathway, thereby promoting BC cell drug resistance. This study may offer novel insights into BC treatment.

## 1. Introduction

Breast cancer (BC) is the main cause of cancer-related mortality in women throughout the world, and the number of BC-caused deaths in 2017 worldwide was 60,728, occupying 14.8% of all cancer deaths among women [1, 2]. Multiple risk factors are implicated in the occurrence of BC, including family history, alcohol consumption, physical inactivity, early menstruation, late menopause or pregnancy, recent use of oral contraceptives and obesity after menopause, never having children, and older age [3, 4]. The symptoms of BC depend on the site of metastasis, of which depression, weariness, insomnia, and pain are the frequent signs [5], and some underestimated symptoms, like persistently feeling cold, dehydration, susceptibility to headache and dizziness, and local and systemic hypoxic effects, should also be given close attention [6]. Adriamycin and docetaxel are commonly applied for BC treatment, but their efficacy is often limited by chemoresistance [7]. Besides, metastasis, drug resistance, and disease recurrence have brought great difficulties to the clinical outcome of BC [8]. Therefore, it is imperative to figure out the molecular mechanisms of BC onset and drug resistance.

Extracellular vesicles (EVs) are bioactive molecular shuttles packaged by proteins, lipids, and nucleic acids, which can modulate the tumor microenvironment (TME) by interacting with adjacent cells [9]. Tumor-derived EVs function as crucial mediators of intercellular communication between tumor cells and normal stromal cells in the local and distant TME, thereby facilitating tumor progression and drug resistance [10]. MicroRNAs (miRNAs) represent the most widely studied molecules in EVs [11]. miRNA has about 22 nucleotides in size, which functions as an antisense RNA to downregulate the expression of target genes at the posttranscriptional level [12]. Evidence has demonstrated that miRNAs circulate in body fluids in a highly stable and acellular form in cancer patients, which may be due to their incorporation in EVs, making them useful as new diagnostic and prognostic markers [13]. Intriguingly, aberrant miRNA expression has also been commonly accepted as a promising biomarker for the drug resistance of BC [14]. Drug-resistant BC cells can release EVs through specific miRNA cell-to-cell metastasis and spread resistance to sensitive cells [15]. Rolf Sokilde et al. have analyzed the BC miRNA microarray GSE131599 and found that 11 miRNAs including miR-887 are notably overexpressed in ER^+^ BC [16]. Lv et al. have shown that miR-887 is highly expressed in BC cell lines, and inhibition of miR-887 enhances the sensitivity of BC patients to 5-Fu treatment [17]. However, whether EVs can affect the drug resistance of BC cells by carrying miR-887-3p is unclear yet. This study investigated the effect of miR-887-3p carried by EVs on the drug resistance of BC, which shall confer novel insights into the clinical management of BC.

## 2. Materials and Methods

### 2.1. Ethics Statement

This study got the approval and supervision of the Ethics Committee of Anhui No. 2 Provincial People's Hospital. Animal experiments were approved by the institutional ethical guidelines. Significant efforts were made to minimize the number of animals and their pain.

### 2.2. Bioinformatics Analysis

The target genes of miRNA were predicted by ENCORI-StarBase (http://starbase.sysu.edu.cn/), RNAInter database (http://www.rna-society.org/rnainter/), and Targetscan (http://www.targetscan.org/vert_71/). The coexpression relationship of genes was searched through Coexpedia database (http://www.coexpedia.org/), and the coexpression score was obtained to further screen the target genes.

### 2.3. BC Cell Lines

Human BC cell lines MDA-MB-231, MCF-7, BT474, and HCC1937 (parental drug-sensitive cells) provided by American Type Culture Collection (ATCC, Manassas, VA, USA) were exposed to gradient concentrations of different drugs (Sigma-Aldrich, Merck KGaA, Darmstadt, Germany), respectively, to generate cisplatin-resistant BT474 (BT474/Cis) cells, doxorubicin-resistant MCF-7 (MCF-7/Dox) cells, and fulvestrant-resistant HCC1937 (HCC1937/Ful) cells. Cells were cultivated in complete modified Eagle's medium with 10% fetal bovine serum.

### 2.4. miRNA Transfection

MDA-MB-231 cells in the logarithmic growth phase were assigned to 3 groups: MDA-MB-231 group (blank control, untransfected cells), MDA-MB-231-NC group (negative control), and MDA-MB-231-inhi group (cells were transfected with miR-887-3p inhibitor). The cells were seeded into the 6-well plates (1 × 10^5^ cells/well). When the cells reached 60% confluence, miR-887-3p inhibitor or NC (GenePharma, Shanghai, China) was transfected into the adherent cells at the concentration of 50 nM using Lipofectamine 2000 (Invitrogen, Carlsbad, CA, USA). After 48 h, the cells were collected for subsequent experiments.

### 2.5. EV Extraction

EVs were isolated and purified with polyethylene glycol- (PEG-) based enrichment and ultracentrifugation as reported previously [18]. A 2 × stock solution of PEG6000 (Sigma-Aldrich) was prepared. MDA-MB-231 cell medium was centrifuged at 500 × g for 5 min and then at 2000 × g for 30 min at 4°C to eliminate cellular debris and large apoptotic bodies. Next, the conditioned medium (CM) was filtered through a 0.22 *μ*m filter (Merck Millipore, Billerica, MA, USA) to clear away microvesicles. Afterwards, an equal amount of prepared 2 × PEG solution was added to the CM and then incubated at 4°C for 12 h while vibrating. The next day, the samples were centrifuged at 3220 × g and 4°C for 1 h and resuspended with particle-free phosphate-buffered saline (PBS). Subsequently, EVs were ultracentrifuged at 110,000 × g and 4°C for 70 min with an Optima XPN-100 ultracentrifuge (Beckman Coulter, Chaska, MN, USA) and then washed to remove contaminated proteins and PEG. Finally, the EVs were resuspended again in particle-free PBS for immediate use or were stored at -80°C. The EVs were identified by a transmission electron microscope (TEM) and nanoparticle tracking analysis (NTA).

### 2.6. Uptake of EVs

MCF-7, HCC1937, and BT474 cells were stained using CellTrace CFSE cell proliferation kits (Invitrogen). EVs were labeled using a PKH26 Fluorescent Cell Linker kit (Sigma-Aldrich). Briefly, 1 mL PKH26 solution (1 : 1000) was mixed with EVs (20 *μ*g protein) for 20 min, and the mixture was washed with PBS and centrifuged at 1000000 × g for 70 min. Then, the PKH26-labeled EVs were added into CFSE-labeled cells for 24 h incubation, and the uptake of EVs was observed under a confocal fluorescence microscope.

### 2.7. Cell Counting Kit-8 (CCK-8) Assay

After drug treatment, cell viability was measured by CCK-8 assay. In detail, cells were seeded into 96-well plates at 3 × 10^3^ cells/well and then exposed to cisplatin (0/0.5/1.0/2.5/5.0/10.0 *μ*M), fulvestrant (0/0.05/0.1/0.5/2.5/5.0 *μ*M), or doxorubicin (0/0.05/0.1/0.5/2.5/5 *μ*M) with increasing concentrations for 3 days. Then, the exposed cells were incubated with 10 *μ*L CCK-8 solution (5 mg/mL, Sigma-Aldrich) for 4 h. Two hours later, each well in the medium was added with 150 *μ*L dimethyl sulfoxide (DMSO, Sigma-Aldrich) to dissolve the formazan crystals. Absorbance value at 490 nm was measured using a microplate reader (Bio-Rad, Hercules, CA, USA) to draw growth curves.

### 2.8. Colony Formation Assay

Cells were resuspended with Roswell Park Memorial Institute- (RPMI-) 1640 culture medium (Gibco, Gaithersburg, MD, USA) in Luria-Bertani culture plates (D0110, Beijing Nobleryder Science and Technology Co., Ltd., Beijing, China). Each cell medium (10 cm) was seeded with 500 cells at 37°C with 5% CO_2_ for two weeks. MCF-7, BT474, and HCC1937 cells were exposed to 2.5 *μ*M doxorubicin, 1 *μ*M cisplatin, or 0.5 *μ*M fulvestrant for 2 h, respectively. After being fixed with 4% paraformaldehyde for 20 min, cells were dyed with crystal violet staining solution for 60 min. Finally, the plates were air-dried and colonies with more than 50 cells were counted under the microscope [19].

### 2.9. Flow Cytometry

An apoptosis assay was performed using the fluorescein isothiocyanate Annexin V Apoptosis Detection Kit (KeyGen, Nanjing, Jiangsu, China). Propidium iodide was combined with Annexin V to examine if cells were viable, apoptotic, or necrotic by a flow cytometer (FACScan®; BD Biosciences, San Jose, CA, USA) equipped with CellQuest software (BD Biosciences). Unstained cells were used as the negative control.

### 2.10. Reverse Transcription Quantitative Polymerase Chain Reaction (RT-qPCR)

The total RNA was extracted from cells using the Trizol kit (Invitrogen), the purity was assessed with an ultraviolet spectrophotometer, and absorbance values at 260 nm (A260) and 280 nm (A280) were obtained. RNA samples that accorded with A260/A280≧1.70 standards were selected for later experiments. After that, RNA was reversely transcribed into cDNA using Revert Aid First Strand cDNA Synthesis Kit (Thermo Fisher Scientific, San Jose, CA, USA), and PCR amplification was then performed. The PCR conditions were 95°C for 60 s, 95°C for 20 s, 58°C for 30 s, and 74°C for 30 s. The amplification primer sequences are exhibited in Table 1.

### 2.11. Dual-Luciferase Reporter Gene Assay

The 3′ untranslated region (3′UTR) mRNA fragment of BTB domain containing 7 (BTBD7) was amplified and cloned into the PmeI and XbaI sites of Pmir Cytomegalovirus (CMV) vector (Promega, Madison, WI, USA). The two constructed vectors were sequenced and named BTBD7-wild-type (wt) and BTBD7-mutant-type (mut) plasmids and then cotransfected with miR-887-3p mimic or mimic control. After 48 h transfection, cells were harvested and the luciferase activity was evaluated with the Dual-Glo Luciferase Assay System (Promega) and a MicroLumatPlus LB96V luminometer (Berthold Technologies, Bad Wildbad, Germany). Relative luciferase activity was calculated as a ratio of the firefly luciferase activity to the Renilla luciferase activity.

### 2.12. Biotinylated RNA Pull-down Assays

Cell lysates were treated with RNase-free DNase I (Sigma-Aldrich) and cultured with a mixture of biotinylated RNA fragments of miR-887-3p (1 *μ*g) and streptavidin-coated magnetic beads (Sigma-Aldrich) at 4°C for 3 h. The RNA was extracted from the captured RNA-RNA complexes for western blot analysis.

### 2.13. Western Blot Analysis

Cells were lysed in ice-cold radioimmunoprecipitation buffer supplemented with protease inhibitor phenylmethylsulfonyl fluoride (1 mM). Equal amounts of proteins (50 *μ*g) in each line were run on 10% sodium dodecyl sulfate-polyacrylamide gel electrophoresis (Bio-Rad) and transferred to polyvinylidene fluoride membranes (Amersham Pharmacia, Piscataway, NJ, USA). Western blots were probed with primary antibodies against BTBD7 (sc-241937, Santa Cruz Biotechnology, Santa Cruz, CA, USA), Notch1 (1 : 1000, ab52627, Abcam, Cambridge, MA, USA), Hes1 (1 : 1000, ab108937, Abcam), CD63 (1 : 1000, ab59479, Abcam), CD81 (1 : 1000, ab79559, Abcam), tumor susceptibility gene 101 (TSG101) (1 : 1000, ab30871, Abcam), Alix (1 : 1000, ab275377, Abcam), GM130 (1 : 20, ab52649, Abcam), and Cyto-C (bs-0013R, Xinyu Biotech Co., Ltd, Shanghai, China) and then incubated with the secondary antibody horseradish peroxidase-labeled goat anti-rabbit immunoglobulin G (IgG) (1 : 2000; A0208; Beyotime Institute of Biotechnology, Shanghai, China). Glyceraldehyde-3-phosphate dehydrogenase (GAPDH) was a loading control for normalization. Immunoblots were visualized using the enhanced chemiluminescence (Amersham Pharmacia) and analyzed with the ImageJ v1.48u software (National Institutes of Health, Bethesda, Maryland, USA).

### 2.14. Animal Studies

A total of 48 BALB/c mice (aged 4-6 weeks; weighing 18 to 25 g) purchased from Guangdong Medical Laboratory Animal Center (Foshan, Guangdong, China) were kept under specific pathogen-free conditions. MCF-7 cells were resuspended with 50 *μ*L PBS and added with 50 *μ*L Matrigel at 5 × 10^6^ cells/mL and subcutaneously injected into each mouse. After bearing the tumors for 7 days, 100 *μ*g EVs extracted from MDA-MB-231 cells (BC+EV group), extracted from miR-887-3p inhibitor-transfected MDA-MB-231 cells (BC+EV-inhi group), or extracted from NC-transfected MDA-MB-231 cells (BC+EV-NC group) were injected into each mouse via a tail vein. Six aliquot injections were administered at 2-day intervals. PBS was used as the vehicle (BC group). The dose of EVs was based on the previous literature [20]. Mice were injected with doxorubicin (10 mg/kg, i.v., push) once on days 12, 15, and 18 after subcutaneous implantation of EVs in MCF-7 cells. The growth of BC xenograft in mice was monitored every 5 days, and 20 days later, tumor growth was monitored every 3 days. At 32 days post-implantation, the mice were euthanized by carbon dioxide asphyxiation.

### 2.15. Immunohistochemical Staining

Tumor sections (5 *μ*m) from xenografts of BC in nude mice were stained using anti-BTBD7 (sc-241937, Santa Cruz Biotechnology) and anti-Ki67 antibody (1 : 50, MIB-1, Immunotech, Marseille, France) at 4°C overnight and then reacted with anti-IgG secondary antibody (1 : 1000; ab6721; Abcam) for 30 min. Then, sections were visualized using 3,3-diaminobezidine (DA1010, Solarbio, Beijing, China). Five fields of view at 200 × magnification were randomly captured for each replicate using an inverted microscope (Nikon, Tokyo, Japan).

### 2.16. Statistical Analysis

All experiments were performed three times. Data were exhibited in the mean ± standard deviation. Statistical analysis was performed with GraphPad Prism 8 software (GraphPad, San Diego, CA, USA). Data were analyzed using the one-way or two-way analysis of variance (ANOVA), followed by Tukey's post hoc test. The *p* value < 0.05 was regarded as statistically significant.

## 3. Results

### 3.1. Extraction and Identification of EVs

**Figure 1:**
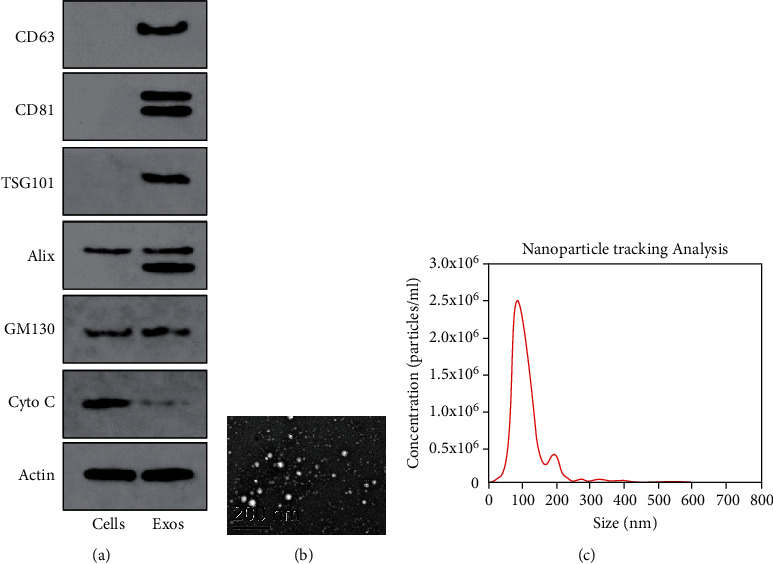
Characterization of MDA-MB-231-derived EVs. (a) Expression of exosomal markers in MDA-MB-231-derived EVs was assessed by western blot analysis. “Cells” mean total cellular protein, and “EVs” present small extracellular vesicles. The exosomal fractions are enriched in tetraspanins (CD63 and CD81) and endosomal markers (Alix and TSG101), but do not contain Golgi (GM130) or mitochondrial (cytochrome C) markers. Actin served as a loading control. (b) TEM was used to observe the morphology of MDA-MB-231-derived EVs at 80,000 × magnification and 120,000 × magnification, showing homogenous, cup-shaped vesicles at 100 nm. Scale bar represents 200 nm in both panels. (c) Nanoparticle tracking analysis was applied to analyze the size of MDA-MB-231 EVs, representing as size vs. concentration. TSG101: tumor susceptibility gene 101; TEM: transmission electron microscope.

EVs were extracted from MDA-MB-231 cells by ultracentrifugation. The biomarker proteins of EVs were determined using western blot analysis, and the results demonstrated that CD63 and CD81 were only expressed in EVs; TSG101 and Alix were highly expressed in EVs than in cell lysates, while GM130 and Cytochrome C was not expressed in EVs (Figure 1(a)). Under the TEM, it was observed that the extracted EVs were uniformly distributed and presented in vesicles of different sizes (Figure 1(b)). The size of the extracted EVs was identified by NTA, and EV particles were mainly distributed at about 100 nm with a concentration of 2.5 × 10^6^ particles/mL (Figure 1(c)). These results indicated that the MDA-MB-231-derived EVs were extracted successfully.

### 3.2. MDA-MB-231-Derived EVs Promoted BC Cell Drug Resistance

**Figure 2:**
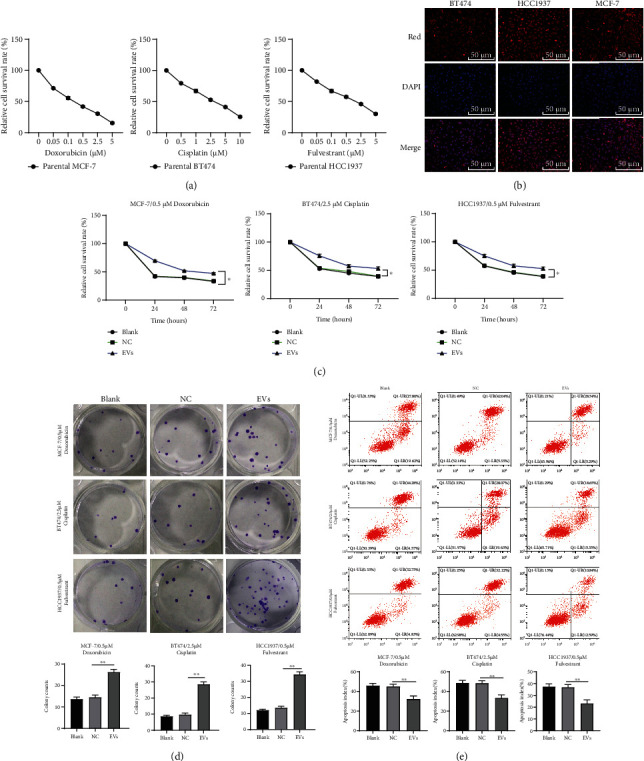
MDA-MB-231-derived EVs promoted BC cell drug resistance. (a) The survival of MCF-7, HCC1937, and BT474 cells in different concentrations of doxorubicin (Dox), cisplatin (Cis), and fulvestrant (Ful) was detected using CCK-8 assay. Then, the BC cells were treated with 15 *μ*g/mL MDA-MB-231-derived EVs (EV-group) or MDA-MB-231 supernatant supplemented with GW4869 (NC group), or PBS as a negative control (blank group). (c) Fluorescence microscope showed the uptake of EVs (15 *μ*g/mL) labeled with the red fluorescent dye PKH26 by MCF-7, BT474, and HCC1937 cells after 24 h of stimulation. Next, MDA-MB-231-derived EV-treated parental or drug-resistant BC cells were exposed to 0.5 *μ*M doxorubicin, 2.5 *μ*M cisplatin, and 0.5 *μ*M fulvestrant, respectively. Then, CCK-8 assay (c), colony formation assay (d), and flow cytometry (e) were performed to determine the effect of MDA-MB-231-derived EVs on BC cell drug resistance. Data are expressed as the mean ± standard deviation; one-way ANOVA and Tukey's multiple comparison test were used to determine statistical significance. ^∗^*p* < 0.05 and^∗∗^*p* < 0.01. Three independent experiments were performed. BC: breast cancer; CCK-8: cell counting kit-8; ANOVA: analysis of variance.

The survival of MCF-7, HCC1937, and BT474 cells in different concentrations of doxorubicin (Dox), cisplatin (Cis), and fulvestrant (Ful) was detected using CCK-8 assay, and the drug concentration of 0.5 *μ*M Dox, 2.5 *μ*M Cis, and 0.5 *μ*M Ful was used for subsequent drug resistance study (Figure 2(a)). Subsequently, the EVs extracted from PKH26-labled MDA-MB-231 cells (15 *μ*g/mL) were added to MCF-7, HCC1937, and BT474 cells for 24-h cultivation. A fluorescence microscope showed that EVs were absorbed by all groups of cells (Figure 2(b)). EV-treated MCF-7, BT474, and HCC1937 cells were treated with 0.5 *μ*M Dox, 2.5 *μ*M Cis, and 0.5 *μ*M Ful, respectively, followed by CCK-8, colony formation, and cell apoptosis detection. The results showed that the addition of EVs notably increased the viability of MCF-7, BT474, and HCC1937 cells under drug treatment and reduced apoptosis (all *p* < 0.05, Figures 2(c)–2(e)). Briefly, MDA-MB-231-derived EVs enhanced BC cell drug resistance.

### 3.3. MDA-MB-231-Derived EVs Carried miR-887-3p into BC Cells

**Figure 3:**
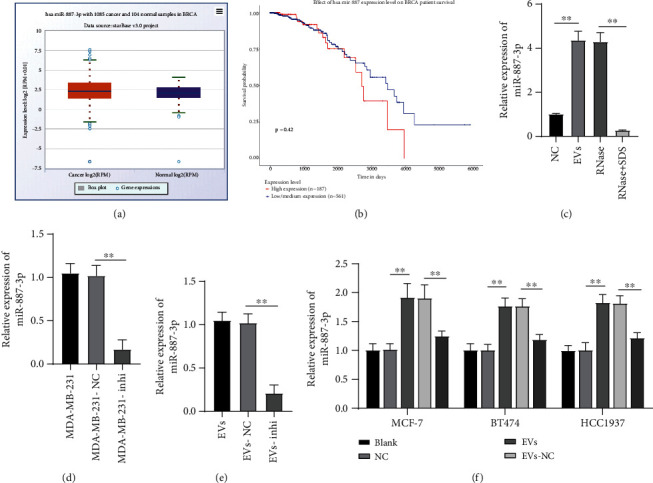
MDA-MB-231-derived EVs carried miR-887-3p into BC cells. ECORI Pan-Cer database analyzed the expression (a) and prognosis (b) of miR-887-3p in BC. EVs were treated with RNase (RNase-group) and RNase+SDS (RNase+SDS-group), respectively. miR-887-3p expression was detected using RT-qPCR (c). Next, miR-Inhibitor was transfected into MDA-MB-231 cells while Mock miR served as a negative control. Then, EVs (EV-NC group and EV-inhi group) were extracted as described in a method. RT-qPCR was performed to determine miR-887-3p expression in MDA-MB-231 cells (d) and EVs (e). MCF-7, BT474, and HCC1937 cells were treated with EVs in different groups, and then RT-qPCR was performed to determine miR-887-3p expression (f). Data were expressed as the mean ± standard deviation. In (f), two-way ANOVA and Tukey's multiple comparison test were used to determine statistical significance, whereas in (c)–(e) one-way ANOVA and Tukey's multiple comparison test were used. ^∗^*p* < 0.05 and^∗∗^*p* < 0.01. Three independent experiments were performed. BC: breast cancer; miR: microRNA; RT-qPCR: reverse transcription quantitative polymerase chain reaction; ANOVA: analysis of variance.

The analysis of BC miRNA microarray (GSE131599) shows that 11 miRNAs including miR-887 are highly expressed in ER^+^ BC [16]. ECORI Pan-Cer database also demonstrated that miR-887-3p was highly expressed in BC (Figure 3(a)) and associated with unfavorable prognosis as shown by the TCGA database (http://ualcan.path.uab.edu/analysis.html) (Figure 3(b)). miR-887-3p expression in EVs was notably higher than that in MDA-MB-231-conditioned medium treated with GW4869 (NC group) (Figure 3(c)). Therefore, we speculated that EVs played a role in chemotherapy resistance of BC by transporting miR-887-3p. After RNase treatment, miR-887-3p expression in EVs did not change significantly, while miR-887-3p expression was significantly reduced after SDS+RNase treatment (all *p* < 0.01, Figure 3(c)), indicating that miR-887-3p was encapsulated in EVs. Moreover, MDA-MB-231 cells were transfected with miR-887-3p inhibitor and then EVs were extracted (EVs-inhi), and miR-887-3p expression in MDA-MB-231 cells and EVs was significantly reduced (all *p* < 0.01, Figures 3(d) and 3(e)). MCF-7, BT474, and HCC1937 cells were treated with EVs-inhi, and the results revealed that miR-887-3p expression in each group of cells was decreased compared with that in the EV group (all *p* < 0.01, Figure 3(f)). Taken together, MDA-MB-231-derived EVs carried miR-887-3p into BC cells.

### 3.4. Inhibition of miR-887-3p in EVs Weakened Drug Resistance and Growth of BC Cells Induced by EVs

**Figure 4:**
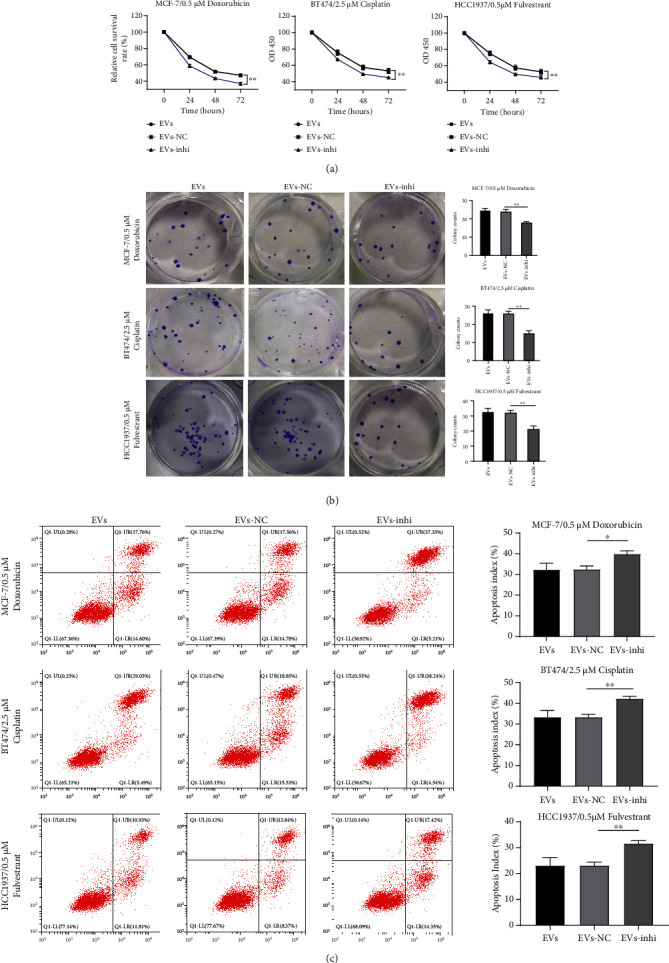
MDA-MB-231-derived EVs carrying miR-887-3p promoted BC cell drug resistance. MDA-MB-231-derived EV-treated BC cells were exposed to 0.5 *μ*M doxorubicin, 2.5 *μ*M cisplatin, and 0.5 *μ*M fulvestrant, respectively. Then, CCK-8 assay (a), colony formation assay (b), and flow cytometry (c) were performed to assess effects of MDA-MB-231-derived EVs carrying miR-887-3p on BC cell drug resistance. Data are exhibited as the mean ± standard deviation. One-way ANOVA and Tukey's multiple comparison test were used to determine statistical significance. ^∗^*p* < 0.05 and^∗∗^*p* < 0.01. Three independent experiments were performed. BC: breast cancer; miR: microRNA; RT-qPCR: reverse transcription quantitative polymerase chain reaction; CCK-8: cell counting kit-8; ANOVA: analysis of variance.

To further verify the role of miR-887-3p carried by MDA-MB-231-derived EVs in drug resistance of BC cells, we added EV-inhi (15 *μ*g/mL) into MCF-7, HCC1937, and BT474 cells for 24 h incubation. Then, EV-treated MCF-7, BT474, and HCC1937 cells were treated with 0.5 *μ*M Dox, 2.5 *μ*M Cis, and 0.5 *μ*M Ful, respectively, followed by cell viability, colony formation ability, and cell apoptosis detection. The results showed that compared with EV-NC, inhibition of miR-887-3p expression in EVs significantly reduced the viability of MCF-7, BT474, and HCC1937 cells under drug treatment and increased apoptosis (all *p* < 0.05, Figures 4(a)–4(c)), suggesting that inhibition of miR-887-3p in EVs weakened drug resistance and growth of BC cells induced by EVs.

### 3.5. miR-887-3p-Targeted BTBD7

**Figure 5:**
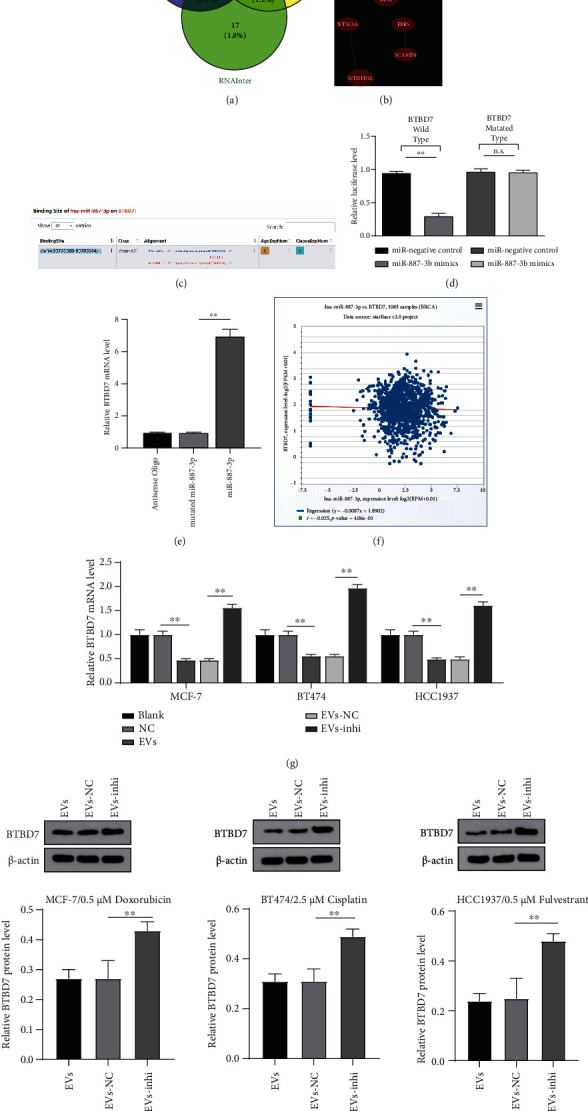
miR-887-3p targeted BTBD7. (a) Venn map of the intersection of miR-887-3p downstream target genes predicted by RNAInter, Targetscan, and ENCORI databases. (b) Coexpression network of candidate genes. (c) miR-887-3p targeting site in BTBD7 3′UTR predicted by Starbase. (d) Luciferase reporter plasmid containing BTBD7-wt or BTBD7-mut was transfected into 293T cells together with miR-887-3p in parallel with an miR-NC plasmid vector. (e) Enrichment of miR-887-3p on the BTBD7 was detected by RNA pull down assay, relative to antisense-oligos. (f) ECORI Pan-Cer assessed the BTBD7 expression in normal tissues and primary tumors. Relative BTBD7 mRNA expression (g) and protein level (h) in MCF-7, BT474, and HCC1937 cells were determined by RT-qPCR and western blot analysis under MDA-MB-231-derived EV treatment. The miR-887-3p was normalized to U6 while the BTBD7 was normalized to GAPDH. Data are expressed as the mean ± standard deviation. One-way ANOVA and Tukey's multiple comparison test were used. ^∗^*p* < 0.05 and^∗∗^*p* < 0.01. Three independent experiments were performed. BC: breast cancer; miR: microRNA; wt: wild type; mut: mutant type; RT-qPCR: reverse transcription quantitative polymerase chain reaction; BTBD7: BTB domain containing 7; 3′UTR: 3′ untranslated region; CCK-8: cell counting kit-8; ANOVA: analysis of variance.

To determine the downstream mechanism of miR-887-3p regulating drug resistance in BC cells, we predicted target genes of miR-887-3p through RNAInter, Targetscan, and ENCORI (Figure 5(a)). The Coexpedia database was used to search the coexpression relationship of genes to further screen target genes. According to the coexpression relationship score, we screened the three target genes MDM4, BTBD7, and MAP3K1 with the highest score (score = 4.677, 2.550, and 2.126) (Figure 5(b)). BTBD7 expression is downregulated in human BC cell lines and tissues, and BTBD7 inhibits the proliferation and invasion/migration of BC cells [21]. Downregulation of BTBD7 can promote apoptosis and increase the sensitivity of NSCLC cells to paclitaxel [22]. Starbase predicted that there is a specific binding site between miR-887-3p and BTBD7 3′UTR (all *p* < 0.05, Figure 5(c)). Based on this sequence, we designed a luciferase reporter plasmid based on CMV, which contained the binding sites of miR-887-3p mimic and miR-negative control with wt or mut BTBD7 3′UTR, respectively. The results showed that miR-887-3p could bind to 3′UTR of BTBD7 specifically (all *p* < 0.05, Figure 5(d)). The RNA pull-down assay further proved the binding relationship between miR-887-3p and BTBD7. The results demonstrated that mutated miR-887-3p could not bind to BTBD7 mRNA, while miR-887-3p could bind to BTBD7 mRNA (all *p* < 0.05, Figure 5(e)). ECORI Pan-Cer database showed that there was a negative correlation between miR-887-3p and BTBD7 in BC patients (Figure 5(f)). Furthermore, we detected the mRNA expression and protein levels of BTBD7 in MCF-7, BT474, and HCC1937 cells after EV treatment. The results showed that miR-887-3p inhibitor-treated EVs upregulated BTBD7 expression (all *p* < 0.05, Figures 5(g) and 5(h)). Briefly, there was a negative regulatory relationship between miR-887-3p and BTBD7.

### 3.6. MDA-MB-231-Derived EVs Carrying miR-887-3p Targeted BTBD7 and Activated the Notch1/Hes1 Signaling Pathway to Promote BC Cell Drug Resistance

**Figure 6:**
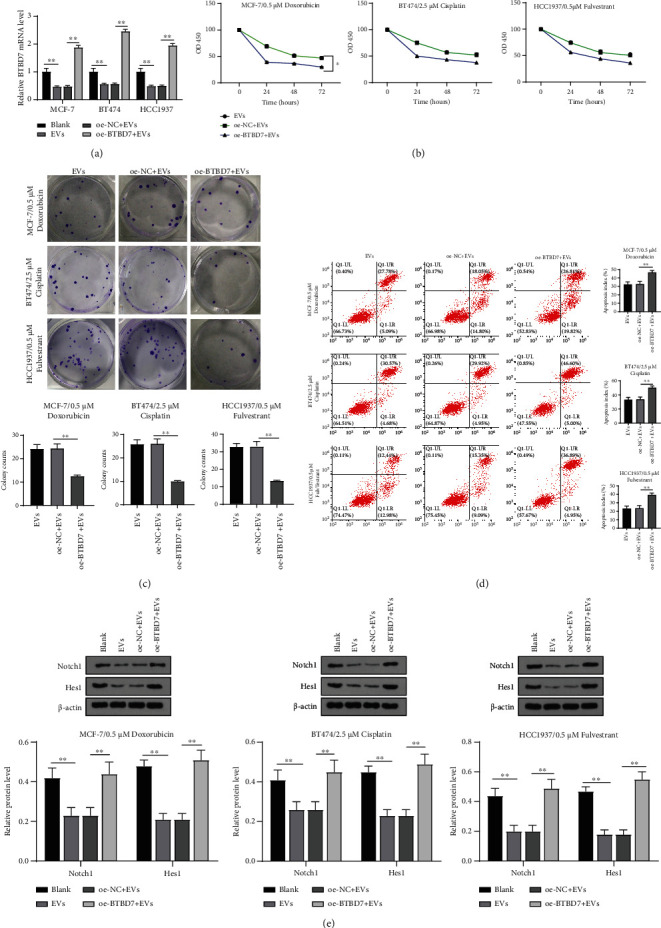
MDA-MB-231-derived EVs carrying miR-887-3p targeted BTBD7 and activated Notch1/Hes1 signaling pathway to promote BC cell drug resistance. MCF-7, HCC1937, and BT474 cells were treated with EVs and transfected with oe-BTBD7. (a) RT-qPCR was performed to determine BTBD7 mRNA expression. CCK-8 assay (b), colony formation assay (c), and flow cytometry (d) were performed to determine the effects of BTBD7 on BC cell drug resistance. Western blot analysis (e) was performed to determine Notch1 and Hes1 protein level in MCF-7, BT474, and HCC1937 cells under MDA-MB-231-derived EV treatments. Data are expressed as the mean ± standard deviation. One-way ANOVA and Tukey's multiple comparison test were used. ^∗^*p* < 0.05 and^∗∗^*p* < 0.01. Three independent experiments were performed. BC: breast cancer; miR: microRNA; BTBD7: BTB domain containing 7; CCK-8: cell counting kit-8; RT-qPCR: reverse transcription quantitative polymerase chain reaction; ANOVA: analysis of variance.

EV-treated MCF-7, HCC1937, and BT474 cells were transfected with oe-BTBD7, and RT-qPCR confirmed the transfection efficiency (*p* < 0.01, Figure 6(a)). Then, the transfected cells were treated with 0.5 *μ*M Dox, 2.5 *μ*M Cis, and 0.5 *μ*M Ful, followed by CCK-8, colony formation, and cell apoptosis detection. The results revealed that compared with EVs, overexpression of BTBD7 significantly reduced the viability of MCF-7, BT474, and HCC1937 cells under drug treatment and increased apoptosis (all *p* < 0.01, Figures 6(b)–6(d)), suggesting that overexpression of BTBD7 reversed drug resistance of BC cells induced by EVs.

Moreover, BTBD7 inhibits BC cell proliferation, invasion, migration, and tumor metastasis by inactivating the Notch1 pathway [21]. The Notch1 pathway can promote drug resistance of cancer cells including gastric cancer cells, ovarian cancer cells, and prostate cancer cells [23–27]. Therefore, we detected the levels of Notch1 pathway-related proteins Notch1 and Hes1 in BC cells, and the results showed that the levels of Notch1/Hes1 were increased after EV treatment, while they were decreased after BTBD7 overexpression (Figure 6(e)). Taken together, MDA-MB-231-derived EVs carrying miR-887-3p targeted BTBD7 and activated the Notch1/Hes1 signaling pathway to promote BC cell drug resistance.

### 3.7. MDA-MB-231-Derived EVs Promoted BC Cell Drug Resistance *In Vivo*

**Figure 7:**
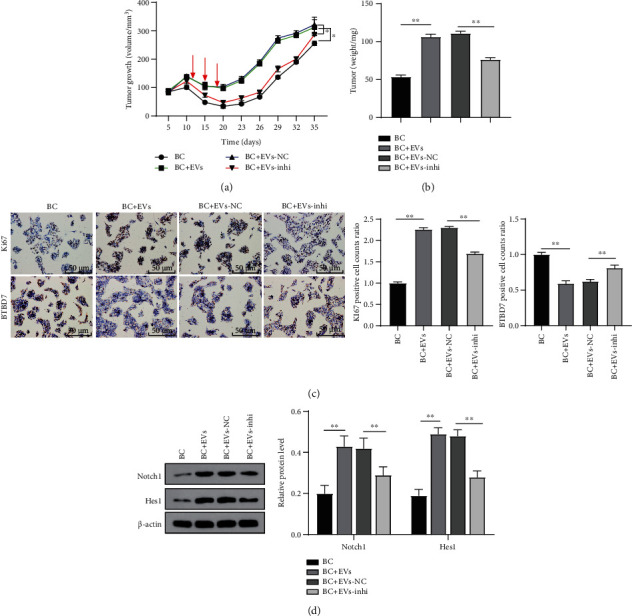
MDA-MB-231-derived EVs promoted BC cell drug resistance *in vivo* by carrying miR-887-3p. Parental MCF-7 and MCF-7/Dox cells stably expressed or transfected were inoculated subcutaneously into BALB/c nude mice at a dose of 5 × 10^6^ per mouse (*n* = 6 in each group). Tumor growth was measured continuously every 5 days, and 20 days later, tumor growth was monitored every 3 days. After bearing the tumors for seven days, 300 mg of EVs was injected into the modeled mice via tail veins. Drug treatments were performed with push of doxorubicin (10 mg/kg) on days 12, 15, and 18 after subcutaneously implantation. At 35 days postimplantation, the mice were euthanized by carbon dioxide asphyxiation. (a) Tumor size. (b) Tumor weight. Tumor sections were obtained and stained with anti-BTBD7 and anti-Ki67 antibodies. (c) Representative views of BTBD7- and Ki67-positive tumor cells and quantification of immunohistochemical staining. (d) Expression of Notch1/Hes1 was detected using western blot. In (a), two-way ANOVA was used to determine statistical significance of quantification of immunohistochemical staining, whereas (b)–(d) one-way ANOVA was used. ^∗^*p* < 0.05. miR: microRNA; BTBD7: BTB domain containing 7; ANOVA: analysis of variance.

To discuss the roles of MDA-MB-231-derived EVs in the growth of MCF-7 cells *in vivo*, we measured the growth and weight of transplanted tumors in nude mice. Compared with doxorubicin treatment alone (BC group), MDA-MB-231-derived EV treatment (BC+EV group) significantly increased tumor growth rate and tumor volume in mice, while miR-887-3p inhibition in EVs (BC+EV-inhi group) weakened the effects (*p* < 0.05, Figures 7(a) and 7(b)). The immunohistochemical results showed that after EV treatment, BTBD7-positive cells were decreased while Ki-67-positive cells were increased in MCF-7-transplanted tumors, which were reversed by inhibition of miR-887-3p (*p* < 0.05, Figure 7(c)). Western blot demonstrated that the levels of Notch1/Hes1 were activated after EV treatment, while inhibition of miR-887-3p reversed such effects (all *p* < 0.01, Figure 7(d)). Briefly, MDA-MB-231-derived EVs promoted BC cell drug resistance *in vivo.*

## 4. Discussion

**Figure 8:**
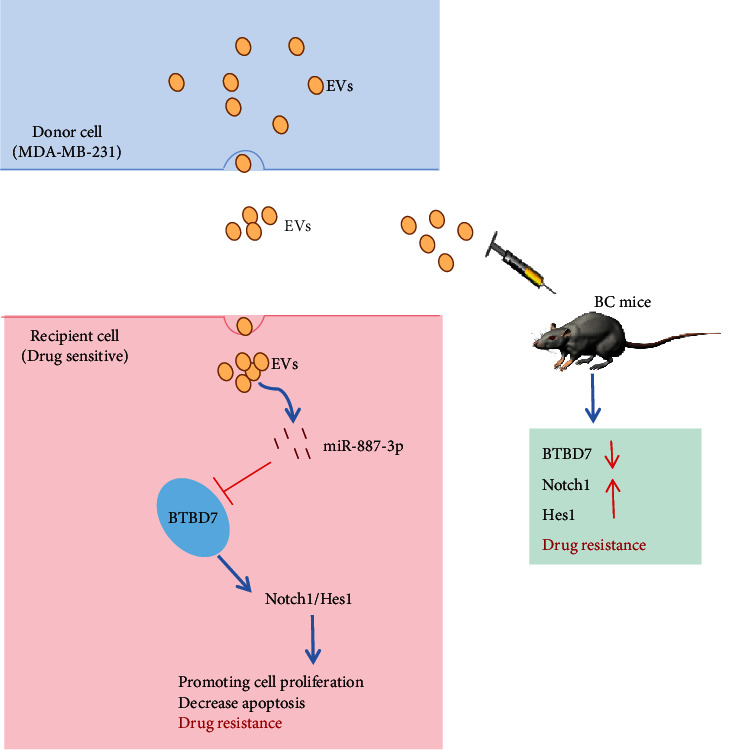
MDA-MB-231-derived EVs can induce drug resistance by carrying miR-887-3p into breast cancer cells to inhibit BTBD7 expression and activate the Notch1/Hes1 signaling pathway.

Although the better treatment and earlier diagnosis of BC have improved in past years, resistance to chemotherapy or radiotherapy, recurrence, and distant metastasis still exist and lead to undesirable prognosis [28, 29]. Strikingly, previous demonstration supported that miRNA-EV delivery decreased the sensitivity of BC cells to doxorubicin [30]. In light of this, we made a hypothesis prior to the experiments that there may be a miRNA-EV delivery between miR-887-3p and MDA-MB-231-derived EVs in BC cell drug resistance. Collectively, such a conclusion could be drawn that miR-887-3p incorporated in MDA-MB-231-derived EVs promoted BC cell drug resistance by targeting BTBD7 and activating the Notch1/Hes1 signaling pathway (Figure 8).

The first major finding in the present study was that MDA-MB-231-derived EVs enhanced BC cell drug resistance. EVs secreted by metastatic tumors promote tumor invasion, inhibit immune responses, and enhance angiogenesis and chemoresistance by delivering RNA between cells [31]. It has recently been described that anticancer drugs strongly increase tumor cell secretion of EVs, facilitating the chemoresistance and posttherapy relapse through signaling pathway activation and inflammation induction [32]. Recently, it reported the miRNA-EV delivery in BC increased EV-induced migration and drug resistance [33]. It was verified that EVs promote angiogenesis, support the growth and expansion of tumors, and transmit anticancer drugs outside the BC cells, leading to drug resistance [34]. Moreover, tumors grow and evolve through a constant crosstalk with the surrounding microenvironment, and emerging evidence indicates that angiogenesis and immunosuppression frequently occur simultaneously in response to this crosstalk [35]. Accordingly, strategies combining antiangiogenic therapy and immunotherapy seem to have the potential to tip the balance of the tumor microenvironment and improve treatment response [36].

As mentioned earlier, docetaxel-resistant BC cells could distribute drug resistance to sensitive BC cells by releasing abundant EVs, which may be partly attributed to the persistent delivery of specific miRNAs [37]. BC cell-derived EVs can induce drug resistance by mediating the expressions of genes and miRNAs concerned with cell proliferation, invasion, migration, and apoptosis [38, 39]. A previous study has investigated the expression of 411 miRNAs related to the drug resistance of 36 BC cell lines and suggested that miRNAs can function as biomarkers of intrinsic drug resistance [40]. Importantly, miR-887 has been demonstrated to be upregulated in BC cells and 5-Fu treatment is enhanced by repressing miR-887 expression [17]. Consistently, this study revealed that miR-887-3p was highly expressed in BC and associated with unfavorable prognosis. We speculated that EVs played a role in chemotherapy resistance of BC by transporting miR-887-3p. Our experiments showed that miR-887-3p expression in EVs did not change significantly after RNase treatment, while it was significantly reduced after SDS+RNase treatment, indicating that miR-887-3p was encapsulated in EVs. Then, MDA-MB-231 cells were transfected with miR-887-3p inhibitor and EVs were extracted (EV-inhi), and miR-887-3p expression in EV-inhi significantly reduced. Moreover, miR-887-3p expression in each group of BC cells was decreased after EV-inhi treatment. Taken together, MDA-MB-231-derived EVs carried miR-887-3p into BC cells. Inhibition of miR-887-3p expression in EVs significantly reduced the viability of MCF-7, BT474, and HCC1937 cells under drug treatment and increased apoptosis, suggesting that inhibition of miR-887-3p weakened drug resistance and growth of BC cells induced by EVs.

Thereafter, we determined the downstream mechanism of miR-887-3p regulating drug resistance in BC cells. BTBD7 is a highly conserved protein, whose major function is to mediate protein-protein interaction [41]. BTBD7 bears various functions in the biological process of eukaryotic cell, including tissue and organ development, protein degradation, and tumor initiation and progression [42]. BTBD7 expression is reduced in BC cells and tissues, and repression of BTBD7 is concerned with positive lymph node status and lymphovascular invasion, while elevation of BTBD7 is related to low BC recurrence [21]. Still, the role of BTBD7 in chemoresistance remains controversial. This study confirmed that there was a negative regulatory relationship between miR-887-3p and BTBD7. Overexpression of BTBD7 significantly reduced the viability of MCF-7, BT474, and HCC1937 cells under drug treatment and increased apoptosis, suggesting that overexpression of BTBD7 reversed drug resistance of BC cells induced by EVs. BTBD7 predicts low recurrence and represses tumor progression by inactivating Notch1 signaling in BC [21]. Notch participates in embryonic development and physiological processes such as cell growth, apoptosis, and differentiation [43]. Notch1 expression is elevated in triple-negative BC and Notch1 activation facilitates triple-negative BC formation [44]. Moreover, activating the Notch1 pathway contributes to enhancing the resistance of gastric cancer cells to chemotherapy [27]. Suppression of Notch1 can reverse the multidrug resistance of gastric cancer cells by inhibiting the expression of multidrug resistance-associated protein Hes [23]. Therefore, we detected the levels of Notch1 pathway-related proteins Notch1 and Hes1 in BC cells, and the results showed that the level of Notch1/Hes1 was increased after EV treatment, while it was decreased after BTBD7 overexpression. Briefly, MDA-MB-231-derived EVs carrying miR-887-3p targeted BTBD7 and activated the Notch1/Hes1 signaling pathway to promote BC cell drug resistance. Moreover, *in vivo* experiments confirmed that MDA-MB-231-derived EVs enhanced BC cell drug resistance via the miR-887-3p/BTBD7/Notch1/Hes1 pathway.

To sum up, MDA-MB-231-derived EVs inhibited BTBD7 expression and activated the Notch1/Hes1 signaling pathway by carrying miR-887-3p into BC cells, thereby enhancing BC cell drug resistance. This study may provide a new understanding of BC treatment in the aspect of cell sensitivity. However, this study failed to verify the prognostic value of miR-887-3p and the role of EVs in BC chemoresistance at the clinical level. Moreover, whether other miRNAs, target genes, and downstream signaling pathways were involved in BC cell drug resistance mediated by EVs remained unclear. In the future, we should investigate the role of upstream lncRNAs in the regulation of miR-887-3p in EVs, or the role of other lncRNAs and miRNAs in EVs in chemotherapy resistance of BC, and other downstream target genes and related signaling pathways that miR-887-3p may regulate.

## Figures and Tables

**Table 1:** Primer sequence.

Gene	Forward 5′-3′	Reverse 5′-3′
miR-887-3p	GGGCGCCACCCGAGGC	AGTGCAGGGTCCGAGGTATT
BTBD7	TGCCAAAGCCTGACTTCTTT	AAGCCAAGGATGCTGCTAAA
U6	GCGCGTCGTGAAGCGTTC	GTGCAGGGTCCGAGGT
GAPDH	AACGACCCCTTCATTGAC	TCCACGACATACTCAGCAC

## Data Availability

The data that support the findings of this study are available from the corresponding author upon reasonable request.

## References

[1] Lin L., Yan L., Liu Y., Yuan F., Li H., Ni J. (2019). Incidence and death in 29 cancer groups in 2017 and trend analysis from 1990 to 2017 from the Global Burden of Disease Study. *Journal of hematology & oncology*.

[2] Lukong K. E., Ogunbolude Y., Kamdem J. P. (2017). Breast cancer in Africa: prevalence, treatment options, herbal medicines, and socioeconomic determinants. *Breast Cancer Research and Treatment*.

[3] Ghoncheh M., Mirzaei M., Salehiniya H. (2015). Incidence and mortality of breast cancer and their relationship with the human development index (HDI) in the world in 2012. *Asian Pacific Journal of Cancer Prevention*.

[4] Torre L. A., Bray F., Siegel R. L., Ferlay J., Lortet-Tieulent J., Jemal A. (2015). Global cancer statistics, 2012. *CA: a Cancer Journal for Clinicians*.

[5] Irvin W., Muss H. B., Mayer D. K. (2011). Symptom management in metastatic breast cancer. *The Oncologist*.

[6] Golubnitschaja O. (2017). Feeling cold and other underestimated symptoms in breast cancer: anecdotes or individual profiles for advanced patient stratification?. *The EPMA Journal*.

[7] Li W. J., Zhong S. L., Wu Y. J. (2013). Systematic expression analysis of genes related to multidrug-resistance in isogenic docetaxel- and adriamycin-resistant breast cancer cell lines. *Molecular Biology Reports*.

[8] Eades G., Wolfson B., Zhang Y., Li Q., Yao Y., Zhou Q. (2015). lincRNA-RoR and miR-145 regulate invasion in triple-negative breast cancer via targeting ARF6. *Molecular Cancer Research*.

[9] Giusti I., di Francesco M., D'Ascenzo S. (2018). Ovarian cancer-derived extracellular vesicles affect normal human fibroblast behavior. *Cancer Biology & Therapy*.

[10] Namee N. M., O'Driscoll L. (2018). Extracellular vesicles and anti-cancer drug resistance. *Biochimica Et Biophysica Acta. Reviews on Cancer*.

[11] Liu T., Zhang Q., Zhang J. (2019). EVmiRNA: a database of miRNA profiling in extracellular vesicles. *Nucleic Acids Research*.

[12] Ghafouri-Fard S., Shoorei H., Taheri M. (2020). miRNA profile in ovarian cancer. *Experimental and Molecular Pathology*.

[13] Kosaka N., Iguchi H., Ochiya T. (2010). Circulating microRNA in body fluid: a new potential biomarker for cancer diagnosis and prognosis. *Cancer Science*.

[14] Jayaraj R., Madhav M. R., Nayagam S. G. (2019). Clinical theragnostic relationship between drug-resistance specific miRNA expressions, chemotherapeutic resistance, and sensitivity in breast cancer: a systematic review and meta-analysis. *Cells*.

[15] Chen W. X., Liu X. M., Lv M. M. (2014). Exosomes from drug-resistant breast cancer cells transmit chemoresistance by a horizontal transfer of microRNAs. *PLoS One*.

[16] Søkilde R., Persson H., Ehinger A. (2019). Refinement of breast cancer molecular classification by miRNA expression profiles. *BMC Genomics*.

[17] Lv Z., Wang S., Zhao W., He N. (2020). MicroRNA analysis of NCI-60 human cancer cells indicates that miR-720 and miR-887 are potential therapeutic biomarkers for breast cancer. *Drug Discoveries & Therapeutics*.

[18] Rider M. A., Hurwitz S. N., Meckes D. G. (2016). ExtraPEG: a polyethylene glycol-based method for enrichment of extracellular vesicles. *Scientific Reports*.

[19] Deng P., Sun M., Zhao W. Y. (2021). Circular RNA circVAPA promotes chemotherapy drug resistance in gastric cancer progression by regulating miR-125b-5p/STAT3 axis. *World Journal of Gastroenterology*.

[20] Qin X., Yu S., Zhou L. (2017). Cisplatin-resistant lung cancer cell-derived exosomes increase cisplatin resistance of recipient cells in exosomal miR-100-5p-dependent manner. *International Journal of Nanomedicine*.

[21] Chen J., Lai Y. H., Ooi S., Song Y., Li L., Liu T. Y. (2020). BTB domain-containing 7 predicts low recurrence and suppresses tumor progression by deactivating Notch1 signaling in breast cancer. *Breast Cancer Research and Treatment*.

[22] Fang L. Z., Zhang J. Q., Liu L. (2017). Silencing of Btbd7 inhibited epithelial-mesenchymal transition and chemoresistance in CD133(+) lung carcinoma A549 cells. *Oncology Research*.

[23] Wang Y. L., Zhang Y., Huang X. Y., Zheng Y. (2018). Reversing effect of NOTCH1 inhibitor LY3039478 on drug-resistance cells SGC7901/DDP of human gastric cancer and its mechanism. *European Review for Medical and Pharmacological Sciences*.

[24] Rice M. A., Hsu E. C., Aslan M., Ghoochani A., Su A., Stoyanova T. (2019). Loss of Notch1 activity inhibits prostate cancer growth and metastasis and sensitizes prostate cancer cells to antiandrogen therapies. *Molecular Cancer Therapeutics*.

[25] Qian X. Q., Tang S. S., Shen Y. M., Chen L. L., Cheng X. D., Wan X. Y. (2020). Notch1 affects chemo-resistance through regulating epithelial-mesenchymal transition (EMT) in epithelial ovarian cancer cells. *International Journal of Medical Sciences*.

[26] Liu X., Luo X., Wu Y. (2018). MicroRNA-34a attenuates paclitaxel resistance in prostate cancer cells via direct suppression of JAG1/Notch1 axis. *Cellular Physiology and Biochemistry*.

[27] Kuai X., Jia L., Yang T. (2020). Trop2 promotes multidrug resistance by regulating Notch1 signaling pathway in gastric cancer cells. *Medical Science Monitor*.

[28] Xu H., Tian Y., Yuan X. (2016). Enrichment of CD44 in basal-type breast cancer correlates with EMT, cancer stem cell gene profile, and prognosis. *Oncotargets and Therapy*.

[29] Masoud V., Pages G. (2017). Targeted therapies in breast cancer: new challenges to fight against resistance. *World journal of clinical oncology*.

[30] Yu D. D., Wu Y., Zhang X. H. (2016). Exosomes from adriamycin-resistant breast cancer cells transmit drug resistance partly by delivering miR-222. *Tumour Biology*.

[31] Hendrix A., Westbroek W., Bracke M., De Wever O. (2010). An ex(o)citing machinery for invasive tumor growth. *Cancer Research*.

[32] Bandari S. K., Purushothaman A., Ramani V. C. (2018). Chemotherapy induces secretion of exosomes loaded with heparanase that degrades extracellular matrix and impacts tumor and host cell behavior. *Matrix Biology*.

[33] Santos J. C., Lima N. D. S., Sarian L. O., Matheu A., Ribeiro M. L., Derchain S. F. M. (2018). Exosome-mediated breast cancer chemoresistance via miR-155 transfer. *Scientific reports*.

[34] Yu D. D., Wu Y., Shen H. Y. (2015). Exosomes in development, metastasis and drug resistance of breast cancer. *Cancer Science*.

[35] Zhao X., Sakamoto S., Maimaiti M., Anzai N., Ichikawa T. (2022). Contribution of LAT1-4F2hc in urological cancers via toll-like receptor and other vital pathways. *Cancers*.

[36] Solimando A. G., Summa S., Vacca A., Ribatti D. (2020). Cancer-associated angiogenesis: the endothelial cell as a checkpoint for immunological patrolling. *Cancers*.

[37] Chen W. X., Cai Y. Q., Lv M. M. (2014). Exosomes from docetaxel-resistant breast cancer cells alter chemosensitivity by delivering microRNAs. *Tumour Biology*.

[38] Si W., Shen J., Zheng H., Fan W. (2019). The role and mechanisms of action of microRNAs in cancer drug resistance. *Clinical epigenetics*.

[39] Ozawa P. M. M., Alkhilaiwi F., Cavalli I. J., Malheiros D., de Souza Fonseca Ribeiro E. M., Cavalli L. R. (2018). Extracellular vesicles from triple-negative breast cancer cells promote proliferation and drug resistance in non-tumorigenic breast cells. *Breast Cancer Research and Treatment*.

[40] Uhr K., Prager-van der Smissen W. J. C., Heine A. A. J. (2019). MicroRNAs as possible indicators of drug sensitivity in breast cancer cell lines. *PLoS One*.

[41] Shu J., Wang L., Han F., Chen Y., Wang S., Luo F. (2019). BTBD7 downregulates E-cadherin and promotes epithelial-mesenchymal transition in lung cancer. *BioMed Research International*.

[42] Cheng D., Qian W., Meng M., Wang Y., Peng J., Xia Q. (2014). Identification and expression profiling of the BTB domain-containing protein gene family in the silkworm, Bombyx mori. *International Journal of Genomics*.

[43] Gharaibeh L., Elmadany N., Alwosaibai K., Alshaer W. (2020). Notch1 in cancer therapy: possible clinical implications and challenges. *Molecular Pharmacology*.

[44] Miao K., Lei J. H., Valecha M. V. (2020). NOTCH1 activation compensates BRCA1 deficiency and promotes triple-negative breast cancer formation. *Nature communications*.

